# Comparing Digital Cognitive Interventions to Active Controls and Usual Care for Mild Cognitive Impairment and Dementia: A Systematic Review and Meta-Analysis

**DOI:** 10.3390/medicina62061162

**Published:** 2026-06-15

**Authors:** Haneul Lee, Youngeun Lim, Seon-Heui Lee

**Affiliations:** 1 Department of Physical Therapy, College of Medical Science, Gachon University, Incheon 21936, Republic of Korea; leehaneul84@gachon.ac.kr (H.L.); ejdnrej618@gachon.ac.kr (Y.L.); 2 Research Institute of AI and Nursing Science, College of Nursing, Gachon University, Incheon 21936, Republic of Korea

**Keywords:** cognitive training, cognitive dysfunction, dementia, systematic review, meta-analysis

## Abstract

*Background and Objectives*: Mild cognitive impairment (MCI) and dementia are prevalent public health challenges with limited pharmacological options for cognitive enhancement. Digital cognitive rehabilitative interventions (DCIs) have emerged as a promising non-pharmacological approach, offering accessibility and personalized strategies. However, their efficacy across diverse populations and contexts remains unclear. This study evaluated the effectiveness of DCIs in improving global cognitive function in individuals with MCI and dementia by comparing them to active controls and usual care. *Materials and Methods*: Ten databases, including Ovid-Medline, Ovid–Embase, Cochrane Library, CINAHL, Web of Science, PsycINFO, KoreaMed, KMbase, RISS, and KISS, were searched for studies published up to May 2025. Global cognitive and executive functions, along with quality of life, were assessed. Meta-analyses using Review Manager version 5.4 were conducted to evaluate global cognitive function improvements, first stratified by comparator group (active control vs. usual care) and further stratified by patient (MCI vs. dementia) and intervention (computer-based vs. virtual reality cognitive training) types. *Results*: This systematic review and meta-analysis analyzed 37 studies. Overall, DCIs improved global cognitive function compared to the control group (SMD = 0.44, 95% CI: 0.18, 0.69). However, subgroup analysis showed no significant effect when DCIs were compared with active controls (SMD = 0.24, 95% CI: −0.35, 0.82). Subgroup analysis showed benefits for individuals with MCI (SMD = 0.43, 95% CI: 0.16, 0.70) but yielded inconclusive results for those with dementia (SMD = 0.95, 95% CI: −0.69, 2.59). Computer-based DCIs were effective (SMD = 0.57, 95% CI: 0.20, 0.93), whereas VR-based interventions had inconsistent outcomes (SMD = 0.32, 95% CI: −0.34, 0.98). *Conclusions*: DCIs may improve cognitive function compared with usual care, particularly in patients with MCI. However, their added benefits overactive cognitive interventions remain uncertain. Further well-designed studies are needed to clarify the relative advantages of DCIs across patient populations and intervention formats.

## 1. Introduction

Mild cognitive impairment (MCI) and dementia are considerable public health issues, with their prevalence rapidly increasing owing to the aging population [1,2]. Individuals with MCI experience noticeable declines in cognitive abilities, such as memory, attention, and executive function, although these deficits do not severely impair daily life [3,4]. MCI is particularly important because it provides a critical window for early intervention, as 10–15% of individuals with MCI progress to dementia each year, and most experience further cognitive decline within 6 years of diagnosis [5]. Dementia, a more advanced stage of cognitive decline, significantly affects the ability of individuals to perform daily activities, leading to a substantial loss of independence [6]. The global prevalence of dementia is increasing, affecting millions of people worldwide and posing a pressing public health challenge [1]. It is associated with profound deficits in memory, language, executive function, and other cognitive domains. Early intervention is critical for slowing or preventing the progression from MCI to dementia, highlighting the importance of effective therapeutic strategies [4].

Despite the importance of addressing MCI and dementia, pharmacological treatments that effectively improve cognitive function or delay disease progression remain limited [7]. This highlights the growing importance of nonpharmacological interventions, which not only help maintain or enhance cognitive function but also reduce the caregiving burden on families and improve the quality of life (QoL) of patients [3,8]. Cognitive intervention therapies encompass distinct approaches, including cognitive stimulation, training, and rehabilitation, which have been widely studied and shown to effectively improve cognitive domains, such as memory, attention, and problem-solving, in both MCI and dementia populations [9]. These interventions aim to enhance cognitive reserve and plasticity, which are crucial for preventing and managing cognitive decline.

Digital cognitive intervention (DCI), a non-pharmacological approach, has gained considerable attention in recent years [10]. By leveraging digital platforms such as mobile applications, virtual reality (VR), and computer-based training, DCIs facilitate cognitive functions through structured and interactive activities [5]. These interventions can be personalized according to the cognitive profile of the user, enabling targeted training that adapts to the individual’s needs. In addition, their remote delivery addresses many logistical barriers, such as limited mobility or access to healthcare facilities [10]. Previous studies have demonstrated their potential to improve global cognitive function and specific neuropsychological domains [5].

A recent systematic review assessed the effects of digital technology interventions on cognitive function in patients with MCI and dementia. Although their findings supported the overall efficacy of DCI, they were limited by the small number of included studies [5], lack of subgroup analyses by comparator [5,11] or patient type [5], and insufficient quantitative synthesis [12]. To address these gaps, this study conducted a comprehensive systematic review and meta-analysis using an expanded dataset to evaluate DCI efficacy across diverse populations. By examining patient-specific characteristics and diverse control conditions, this study aimed to provide insights into how DCI can be tailored to optimize therapeutic outcomes for individuals with varying cognitive profiles.

Therefore, this systematic review and meta-analysis aimed to evaluate the efficacy of DCIs in improving cognitive function in individuals with MCI and dementia based on patient-controlled conditions. Furthermore, subgroup analyses were conducted to explore variations in efficacy based on patient and intervention type, providing a more detailed understanding of how DCIs can be optimized for different populations and contexts. We hypothesized that DCIs would improve cognitive function in individuals with MCI and dementia, with potentially different effects according to patient and intervention type.

## 2. Materials and Methods

### 2.1. Design

This systematic review and meta-analysis was conducted in accordance with the Cochrane Handbook for Systematic Reviews of Interventions [11] and the Preferred Reporting Items for Systematic Reviews and Meta-Analyses Statement (PRISMA) guidelines [12]. The review protocol was registered in the International Prospective Register of Systematic Reviews (PROSPERO) (CRD42024544955) to reduce reporting bias and ensure a comprehensive outcome assessment.

### 2.2. Data Sources and Search Strategy

A comprehensive search was conducted across 10 databases: Ovid-Medline, Ovid–Embase, Cochrane Library, CINAHL, Web of Science, PsycINFO, KoreaMed, KMbase, RISS, and KISS, covering publications up to May 2025. Grey literature was also searched through trial registries, including ClinicalTrials.gov, to identify unpublished or ongoing studies. Two independent reviewers (H. Lee and Y. Lim) searched, collected, and organized the relevant studies using EndNote 20.5. Keywords were selected based on database-specific terminology, including Medical Subject Headings, Emtree terms, and relevant text words. The following keywords were used: (“cognitive dysfunction” OR (“cognitive” AND (“dysfunction*” OR “impairment*” OR “disorder*” OR “deterioration*”))) AND (“dementia” OR “Alzheimer*” OR “mild cognitive impairment”) AND (“telemedicine” OR “telerehabilitation” OR “exergaming” OR “digital health” OR “digital therap*” OR “digital intervention”). A detailed description of the search strategy is provided in Supplementary S1.

### 2.3. Selection Criteria

In this study, the eligibility criteria were based on clinical questions using the PICO framework (Table 1) [12]. This systematic review included randomized control trials (RCTs) and non-randomized studies involving older adults with MCI and dementia. Studies were eligible if they assessed digital interventions targeting an increase in cognitive function compared with an appropriate comparator. In this review, DCIs were defined as structured digital programs designed to improve or maintain cognitive function through cognitive training, cognitive stimulation, or cognitive rehabilitation tasks. Eligible delivery formats included mobile application-, computer/tablet/web-based, and VR-based programs, provided that they included an explicit cognitive intervention component. Digital tools used only for assessment, monitoring, education, communication, or general physical activity without a defined cognitive component were not considered eligible DCIs. The effectiveness of these interventions was evaluated by comparing outcomes, such as global cognitive function, executive function, and QoL, with those of the control groups, including active controls and usual care. “Active controls” were defined as therapeutic interventions aimed at improving cognitive function without using digital tools, while “usual care” referred to standard care, sham digital devices not intended for cognitive function, or no treatment. In addition, only original articles were considered; non-human studies, non-original articles, reviews, observational studies, pre-post studies without a control group, and study protocols were excluded. Study selection was finalized through a discussion. Any disagreements or discrepancies were addressed through consultation with a third reviewer (S-H. Lee).

### 2.4. Data Extraction

Relevant data were extracted and recorded using a standardized data extraction form. This information includes details such as the author, publication year, country, study design, type of patient (MCI or dementia), sample size, age, sex, type of digital intervention, and control group treatments. When data were missing, the researchers contacted the authors via e-mail to request the missing information. If no response was received, the study was included in the systematic review but excluded from the meta-analysis, with missing data noted as being unavailable. The primary outcome for global cognitive function was assessed using the Montreal Cognitive Assessment and Mini-Mental State Examination. Outcomes for executive function improvement included the Trail-Making Test B, the Wechsler Memory Scale-Spatial Span subtest, Executive Interview 25, and the Korean version of the Executive Function Performance Test. QoL was evaluated using the QoL in Alzheimer’s Disease scale. Two independent reviewers conducted an initial screening of the titles and abstracts, followed by a full-text review to select studies that met the eligibility criteria.

### 2.5. Quality Assessment

The quality of the studies was assessed by two reviewers (H. Lee and S-H. Lee) using version 1 of the Cochrane Risk of Bias tool (RoB-1) for RCTs. Any disagreements were resolved through discussion with a third reviewer (Y. Lim). This assessment covered six domains of potential bias: random sequence generation, allocation concealment, blinding of participants and personnel, blinding of outcome assessments, incomplete outcome data, and selective reporting. Each domain was rated as having a ‘low,’ ‘high,’ or ‘unclear’ risk of bias [13].

The quality of evidence was evaluated using the Grading of Recommendations, Assessment, Development, and Evaluation (GRADE) approach. The level of evidence was downgraded by 1 or 2 points from high quality, based on five criteria [14]. The criteria were the risk of bias, inconsistency (*I*^2^: ≥50 or ≥75), indirectness, imprecision (total number of participants: <300), and potential publication bias [14].

### 2.6. Data Synthesis and Analysis

Data were analyzed using Review Manager (RevMan) version 5.4 to quantitatively synthesize the outcomes and assess the effects of the interventions. Continuous outcomes were reported as mean differences (MD) or standardized MD (SMD) with 95% confidence intervals (CIs). The SMD was applied when continuous data were reported on different scales but measured the same concept. When studies reported 95% confidence intervals instead of standard deviations (SD), SDs were calculated using the methods implemented in RevMan software to enable inclusion in the meta-analysis. The median and interquartile ranges were converted to mean ± standard deviation (SD) when necessary [15]. If the actual mean change was not provided, SD was calculated using a validated formula [11].

Statistical heterogeneity between studies was assessed using the chi-square test (*p* < 0.10), and the *I*^2^ statistic quantified the extent of heterogeneity. Given the expected clinical and methodological heterogeneity in participant characteristics, intervention protocols, and comparator interventions, a random-effects model was used for all meta-analyses. Subgroup meta-analyses were performed to address heterogeneity related to variations in comparator interventions, patients, or intervention types. Publication bias was evaluated using a funnel plot when at least 10 studies were available. All results were considered statistically significant at *p* < 0.05. Sensitivity analysis was performed by excluding several studies to assess the robustness of the pooled results.

## 3. Results

### 3.1. Search Results

**Figure 1:**
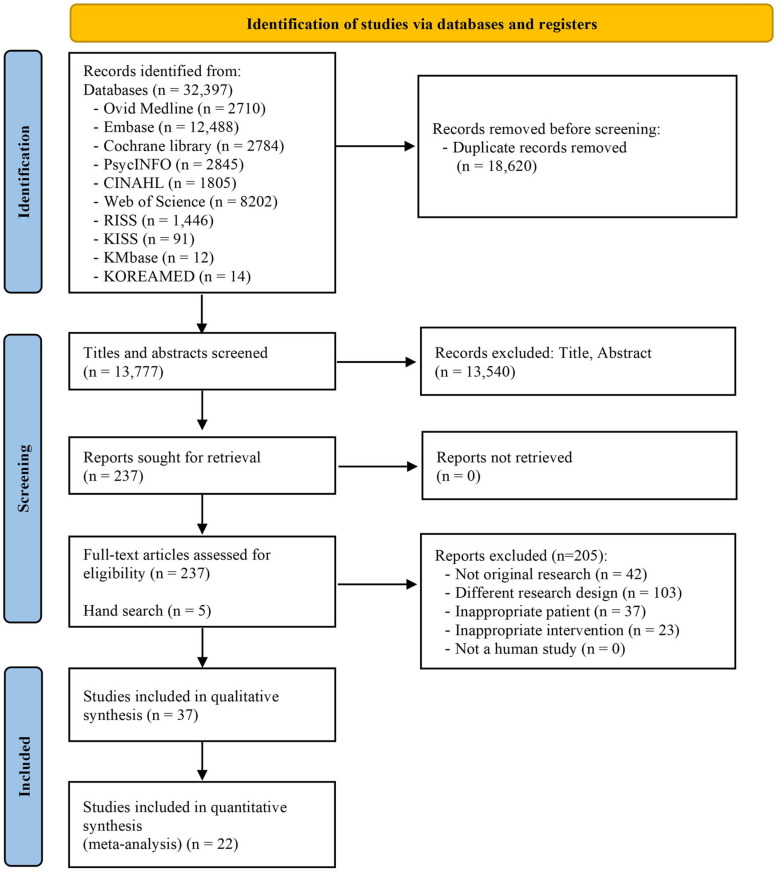
Preferred Reporting Items for Systematic Reviews and Meta-Analyses flow diagram.

In total, 32,397 studies were initially identified through database and manual searching. After removing 18,620 duplicates using the EndNote deduplication function and a preliminary review by the researchers, the titles and abstracts were screened, leading to the exclusion of 13,540 studies from the review. In total, 237 studies were screened and identified based on the eligibility criteria. Finally, 37 studies were included in the qualitative analysis and 22 in the quantitative analysis (meta-analysis) (Figure 1). Data from three studies required conversion from reported 95% confidence intervals to SDs for inclusion in the meta-analysis [16,17,18]. Fifteen studies were excluded from meta-analysis because of insufficient data.

### 3.2. Study Characteristics

Table 2 summarizes the 37 studies identified through database searches conducted up to May 2025. No studies from grey literature sources were included because of insufficient methodological and outcome information. The included studies were published between January 2014 and April 2024. Of these, 36 were RCTs and one was a non-RCT. Eighteen studies were conducted in Europe [1,2,3,4,16,17,18,19,20,21,22,23,24,25,26,27,28,29], 16 in the Asia–Pacific region [6,7,8,30,31,32,33,34,35,36,37,38,39,40,41,42], two in North America [43,44], and one in the Middle East [45].

In total, 2223 participants were included, with individual study sizes ranging from 16 to 158. The follow-up periods varied widely, ranging from 1 (with sessions lasting 20–30 min) to 6 months, with most studies conducting follow-ups for 2–3 months. Among the included studies, 26 focused exclusively on participants with MCI [1,3,4,6,7,8,16,17,18,19,20,21,22,23,30,31,32,33,34,35,36,37,38,39,43,45], nine targeted individuals with dementia [2,24,25,26,27,28,40,41,44], and two included mixed populations comprising participants with MCI and dementia [29,42]. Ten studies utilized virtual reality (VR)-based systems for DCI [4,6,16,23,32,33,35,37,44], while the remaining 27 employed non-VR software-based interventions delivered via computers, tablets, or mobile devices [1,2,3,7,8,17,18,19,20,21,22,24,25,26,27,28,29,30,31,36,38,39,40,41,42,43,45].

### 3.3. Quality Assessment

**Figure 2:**
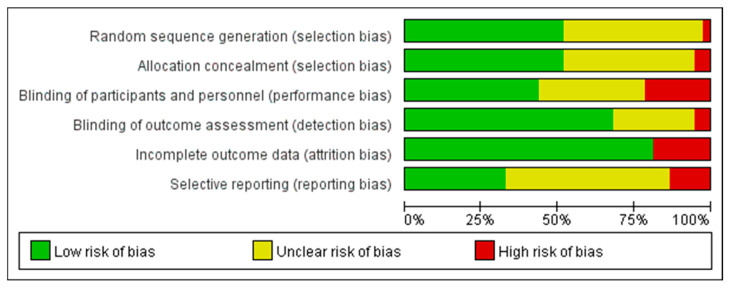
Risk of bias graph.

Thirty-seven studies were evaluated using the RoB-1 (Figure 2). Nineteen RCTs were assessed as having a low risk of bias because they reported the methods of random sequence generation, whereas 17 studies did not provide detailed methods of randomization. Appropriate allocation concealment was performed in 19 studies. In addition, 16 studies had a low risk of bias for participant blinding, and outcome assessment blinding was performed in 25 studies. However, seven studies provided incomplete outcome data, and 20 studies were assessed as unclear owing to the absence of a protocol. The quality of evidence, assessed using the GRADE method, is shown in Supplementary S2.

### 3.4. Meta-Analysis of DCI Efficacy

#### DCI Efficacy by Comparator Groups

**Figure 3:**
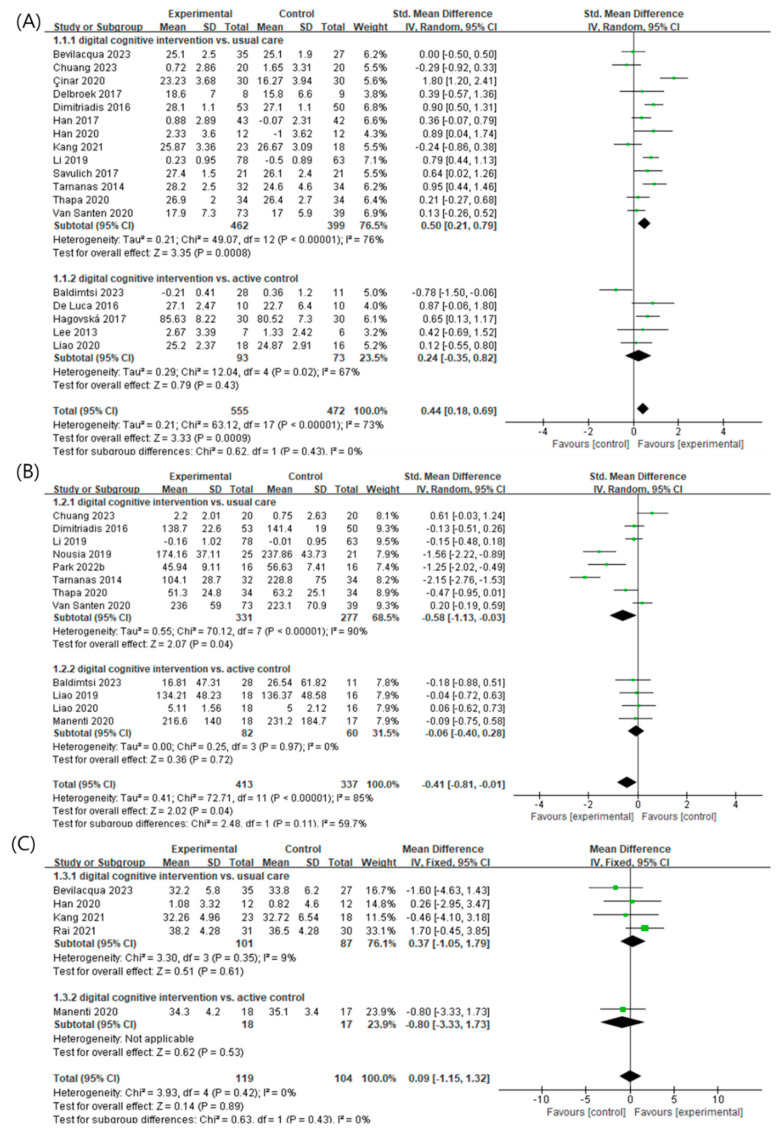
Forest plots of (**A**) global cognitive function [1,4,6,7,8,18,20,21,22,23,25,26,28,30,34,35,36,41], (**B**) executive function [3,4,16,22,23,25,30,33,34,35,36,37], and (**C**) quality of life by types of controls [1,6,7,16,24].

The pooled data from 18 studies demonstrated a significant improvement in global cognitive function in the experimental group compared with the control group (SMD = 0.44, 95% CI [0.18, 0.69], *I*^2^ = 73%, τ^2^ = 0.21) (Figure 3A). In the ‘usual care group’, a statistically significant improvement was observed in global cognitive function with DCI compared to usual care. However, the quality of evidence was rated low owing to the risk of bias and serious inconsistencies (SMD = 0.50, 95% CI [0.21, 0.79], *I*^2^ = 76%, τ^2^ = 0.21) (Figure 3A). The funnel plot showed a symmetric distribution, and Egger’s regression test did not indicate a significant publication bias (intercept = 3.63, *p* = 0.22), suggesting that the observed asymmetry was not statistically significant (Supplementary S3). Sensitivity analysis excluding five exergaming studies showed no substantial change in the pooled effect size, supporting the robustness of the findings (SMD = 0.58, 95% CI [0.15, 1.01], *I*^2^ = 83%, τ^2^ = 0.31). Conversely, in the ‘active control’ group, which included four studies, the pooled data showed no significant difference between DCIs and non-DCIs. The quality of evidence was rated as low owing to serious inconsistency and imprecision (SMD = 0.24, 95% CI [−0.35, 0.82], *I*^2^ = 67% τ^2^ = 0.29) (Figure 3A).

The analysis of 12 studies assessing the impact of DCIs on executive function showed a significant improvement in the experimental group, though with substantial heterogeneity (SMD = −0.41, 95% CI [−0.81, −0.01], *I*^2^ = 85%, τ^2^ = 0.41) (Figure 3B). Subgroup analyses revealed differences in efficacy between the usual care and active control conditions. In the usual care group, the pooled analysis indicated a significant trend favoring the experimental group (SMD = −0.58, 95% CI [−1.13, −0.03], *I*^2^ = 90%, τ^2^ = 0.55), although the quality of evidence was rated as very low owing to serious inconsistency and imprecision (Figure 3B). In contrast, active controls revealed no significant differences between DCIs and non-DCIs, with moderate certainty owing to serious imprecision (SMD = −0.06, 95% CI [−0.40, 0.28], *I*^2^ = 0%, τ^2^ = 0.00) (Figure 3B). This indicates that DCIs have outcomes similar to those of traditional cognitive training in this context. Typically, DCIs showed potential for enhancing executive function compared with usual care.

A meta-analysis of five studies evaluating QoL using the QoL-AD scale revealed no significant differences between the two groups (SMD = 0.09, 95% CI [−1.15, 1.32], *I*^2^ = 0%, τ^2^ = 0.00) (Figure 3C). The usual care group demonstrated a non-significant improvement favoring the experimental group; the quality of evidence was rated as low owing to a serious risk of bias and inconsistency (SMD = 0.37, 95% CI [−1.05, 1.79], *I*^2^ = 9%, τ^2^ = 0.23) (Figure 3C). This indicates a minimal, statistically nonsignificant trend toward better QoL in the experimental group than in the usual care group. Similarly, the active control group showed no significant difference between the experimental and control groups, with low certainty of the quality of evidence owing to serious inconsistency and imprecision (SMD = −0.80, 95% CI [−3.33, 1.73]) (Figure 3C).

### 3.5. Sub-Group Analysis

A subgroup analysis was conducted on studies in which the control group received usual care to minimize heterogeneity, stratifying the results by patient (MCI vs. dementia) and intervention type (computer-based vs. VR-based cognitive training). Studies including mixed populations of dementia and MCI were excluded from subgroup analysis because they could not be clearly classified into either subgroup [32,43]. Studies with active control groups were excluded from the subgroup analysis because of their small sample sizes.

#### 3.5.1. DCI Efficacy by Patient Type

**Figure 4:**
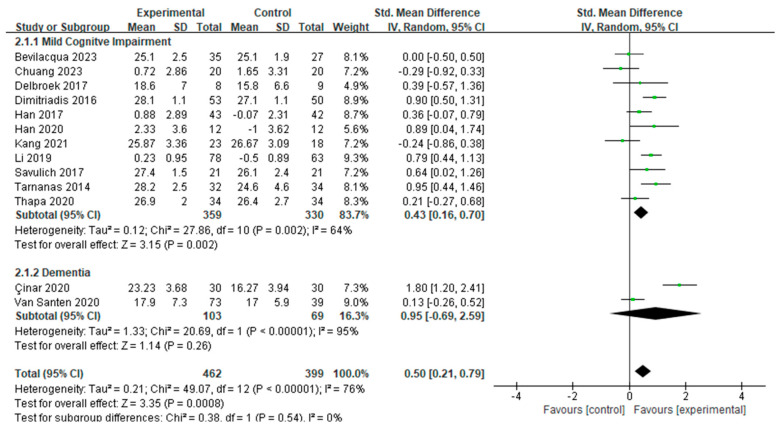
Forest plots of global cognitive function by patient type [1,6,7,8,18,21,22,23,25,26,30,35,36].

Subgroup analysis revealed significant improvements in participants with MCI (SMD = 0.43, 95% CI [0.16, 0.70], *I*^2^ = 64%, τ^2^ = 0.12), indicating moderate heterogeneity (Figure 4). The funnel plot showed a symmetric distribution, and Egger’s regression test did not suggest a significant publication bias (intercept = −0.38, *p* = 0.83), implying that selective reporting was unlikely to have substantially influenced the observed effect sizes. In contrast, the effect of interventions on individuals with dementia was not statistically significant (SMD = 0.95, 95% CI [−0.69, 2.59], *I*^2^ = 95%, τ^2^ = 1.33), with considerable heterogeneity and only two included studies (Figure 4). These findings suggest that, although DCIs effectively enhance global cognitive function in individuals with MCI, their efficacy in patients with dementia remains uncertain and should be interpreted cautiously.

#### 3.5.2. DCI Efficacy by Intervention Types

**Figure 5:**
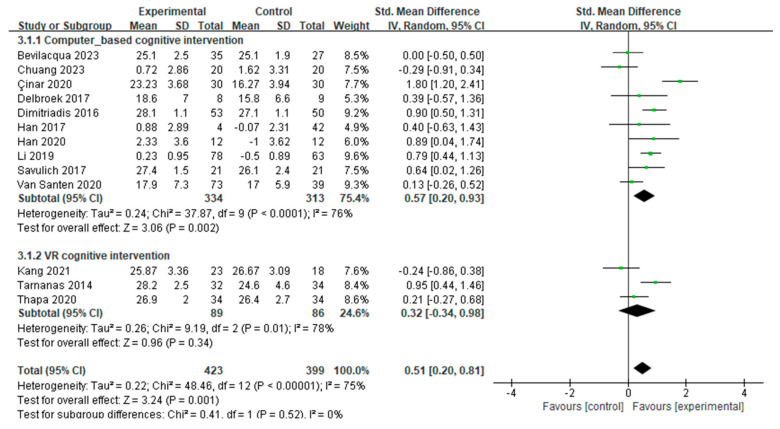
Forest plots of global cognitive function by intervention type [1,6,7,8,18,21,22,23,25,26,30,35,36].

Subgroup analysis revealed significant improvements in computer-based cognitive interventions (SMD = 0.55, 95% CI [0.21, 0.90], *I*^2^ = 77%, τ^2^ = 0.22), indicating substantial heterogeneity (Figure 5). Egger’s regression test did not suggest significant publication bias (intercept = 1.05, *p* = 0.64), implying that selective reporting was unlikely to have substantially influenced the observed effect sizes. Zhu et al., a quasi-experimental study, also reported that computer-based exergaming improved global cognitive function compared with the control group [40]. Three studies involving computer-based exergaming were excluded for sensitivity analysis, and the results remained significant, suggesting the robustness of the findings (SMD = 0.57, 95% CI [0.12, 1.01], *I*^2^ = 84%, τ^2^ = 0.30) [7,10,23]. In contrast, the effect of VR-based cognitive training was not statistically significant (SMD = 0.32, 95% CI [−0.34, 0.98], *I*^2^ = 78%, τ^2^ = 0.26), with considerable heterogeneity (Figure 5). These results suggest that although DCIs effectively enhance global cognitive function in individuals with MCI, their efficacy in patients with dementia remains uncertain.

### 3.6. Usability of DCIs

Despite the growing evidence supporting the efficacy of DCIs, their usability remains underexplored in existing studies. Of the 37 studies included in this review, only four explicitly assessed their usability. For individuals with MCI, the overall usability ratings were generally positive, with most interventions scoring above the acceptable threshold on the System Usability Scale. Participants reported good usability across various DCIs, including telerehabilitation and VR-based systems, with minimal obstacles in engagement and interaction. Although some studies noted minor challenges, such as the need for occasional technical support, the overall findings suggest that digital interventions are well-received and accessible for individuals with MCI [1,16,19]. For individuals with dementia, Rai et al. (2021) examined iCST app usability and found that caregivers rated the system more favorably than patients, despite both groups providing positive feedback on its design and usability [24]. However, frequent use was limited because of the perceived lack of relevance and variety in activities, suggesting that personalization is crucial for sustained engagement in dementia populations [24].

## 4. Discussion

This systematic review and meta-analysis supports the use of digital interventions as a promising approach for addressing cognitive decline in MCI and dementia. Our findings suggest that the effects of DCIs may differ according to patient population and comparator type.

The key findings revealed that DCIs significantly improved global cognitive function in individuals with MCI compared to usual care, consistent with the existing literature suggesting that neuroplasticity present in the early stages of cognitive decline may facilitate responsiveness to targeted interventions [46]. However, the evidence for individuals with dementia was inconclusive because the subgroup analysis was based on a very small number of studies with substantial heterogeneity and wide confidence intervals. These results suggest that DCIs may have greater potential as early interventions for individuals with MCI, but they do not allow firm conclusions regarding their effectiveness in dementia.

### 4.1. Effects of DCI by Comparator Groups

An important aspect of this meta-analysis was the comparison between experimental DCI groups, active control groups (typically non-DCI), and usual care. DCIs demonstrated significant improvements over usual care, suggesting that structured digital cognitive programs may provide benefits compared with standard practices that lack structured cognitive intervention components. However, these findings should be interpreted in relation to the type and intensity of the comparator used. DCIs showed significant improvements compared with usual care but not compared with active controls. However, the nonsignificant finding in the active control comparison should not be interpreted as evidence of equivalence or comparable efficacy. This comparison included only four studies with a small total sample size and wide confidence intervals, indicating insufficient statistical power to determine whether DCIs are superior, equivalent, or inferior to active control interventions. Therefore, future studies should clarify whether the digital format itself provides added cognitive benefits beyond those of active non-digital cognitive interventions. Even when cognitive effects are similar, DCIs may still offer practical advantages in terms of scalability, remote accessibility, and adaptability to individual needs.

Digital interventions may also offer practical advantages, such as enhanced engagement through gamification, real-time feedback, and adaptive difficulty levels, scalable delivery, and remote accessibility [12]. These features could improve adherence and overall user experience, particularly when access to in-person cognitive rehabilitation is limited. However, further studies are needed to determine whether these digital features translate into superior cognitive outcomes compared with active non-digital interventions.

### 4.2. Effects of DCI for MCI but Not Dementia

Subgroup analysis suggested that DCIs were associated with significant improvements in global cognitive function among individuals with MCI. This finding supports the potential role of DCIs as early interventions for cognitive decline. In contrast, the dementia subgroup did not show a statistically significant effect; however, this result should be interpreted with substantial caution because it was based on only two studies with considerable heterogeneity and wide CIs. Therefore, this analysis does not provide sufficient evidence to draw meaningful or generalizable conclusions regarding the effectiveness of DCIs in individuals with dementia. Given the limited and heterogeneous evidence, further studies are needed to determine how DCIs can be adapted for individuals with dementia. Future research should consider disease severity, usability, adherence, caregiver support, and intervention intensity when evaluating DCIs in this population.

These findings suggest that DCI programs may need to be tailored to the needs and capabilities of different patient groups. For individuals with MCI, cognitive training and stimulation may be appropriate targets, whereas for individuals with dementia, future studies should examine whether DCIs are more effective when combined with support for daily functioning, emotional well-being, caregiver involvement, and QoL.

### 4.3. Effects of DCI by Intervention Type

Subgroup analysis suggested that computer-based cognitive interventions improved global cognitive function, whereas VR-based training did not show significant effects. However, these findings should be interpreted cautiously because both intervention types exhibited high heterogeneity, reflecting variability in study design, participant characteristics, and intervention protocols. One possible explanation for this discrepancy is the cognitive and perceptual demands of VR technology. VR environments often require complex visuospatial processing, motor coordination, and sustained attention, which may pose challenges for individuals with cognitive dysfunctions [47]. Patients with MCI or dementia may struggle with immersion, navigation, or task adherence, reducing engagement and limiting the effectiveness of VR-based interventions. This aligns with previous studies suggesting that although VR can be engaging, its usability for cognitively impaired populations remains a concern [48].

In addition, the substantial heterogeneity observed in both intervention types suggests variability in the study design, participant characteristics, and intervention protocols. Further research is needed to explore whether specific adaptations, such as simplified VR interfaces or guided training or caregiver support, can enhance accessibility and effectiveness of VR-based intervention. Although computer-based cognitive interventions appear promising, the effectiveness of VR-based cognitive training remains uncertain and requires further investigation.

### 4.4. Challenges of DCI

Despite the promising outcomes of DCIs, several challenges remain regarding their widespread adoption and implementation. Accessibility and usability issues remain substantial concerns for older adults with cognitive impairments, as many face challenges in navigating complex digital interfaces or lack prior exposure to technology, which limits their engagement and potential benefits [49]. Our findings show that although most interventions for MCI receive favorable usability ratings [1,16], some users require technical support, indicating that even well-designed systems may present challenges [19]. In contrast, usability perceptions for individuals with dementia were more varied, with caregivers finding the systems more useful than patients [24]. This discrepancy suggests that individuals with advanced cognitive decline may require additional support to sustain engagement with DCIs.

Despite the importance of usability in digital interventions, only a few studies in our review explicitly assessed this factor. This highlights a research gap and emphasizes the need for a more systematic evaluation to ensure that DCIs are accessible and user-friendly for a broader population. Furthermore, disparities in digital literacy and access contribute to a growing digital divide, disproportionately affecting socioeconomically disadvantaged individuals and those living in rural areas. Beyond usability concerns, data privacy and security remain critical issues, as many digital interventions require personal health data collection and cloud-based processing, raising ethical and regulatory challenges [50]. To make DCIs more effective and accessible, usability, accessibility, and digital literacy must be considered in their design. Improving these aspects can help ensure that more individuals benefit from these interventions and engage with them over the long-term.

### 4.5. Implications for Clinical Practice

These findings may have substantial implications for clinical practice. Although digital interventions are accessible and scalable, they are most effective when integrated into a broader therapeutic framework to complement traditional cognitive rehabilitation approaches. Individuals with MCI can improve their cognitive health and potentially delay dementia progression. However, for individuals with dementia, the indispensable role of caregivers must be emphasized, as their support enhances adherence and engagement while providing critical emotional and social support.

Enhancing adherence is a critical factor in maximizing the benefits of DCIs. Similar to home-based rehabilitation models [51], structured adherence-supportive strategies, including caregiver involvement, adaptive training difficulty, and real-time progress tracking, are essential for sustained engagement. Baquero et al. found that adherence to DCI was strongly linked to executive function, working memory, and cognitive flexibility. Their findings underscore the need for tailored interventions that accommodate cognitive variability and ensure that individuals with lower cognitive flexibility receive additional support to enhance their engagement [29].

Moreover, DCIs may be integrated into existing clinical and community-based cognitive health programs to improve cognitive health. For example, healthcare institutions may implement structured DCI as part of MCI rehabilitation programs, whereas home-based digital interventions may offer flexible and scalable options for individuals with mobility limitations, ensuring continuous access to cognitive training. The ability to remotely monitor progress and adapt intervention content may further support the practical application of these interventions in diverse healthcare settings.

### 4.6. Limitations and Further Research

This study had some limitations. First, the included studies exhibited considerable heterogeneity in the intervention design, patient population, and outcome measures, which may have limited the generalizability of the findings. Variations in the duration, frequency, and type of DCIs likely contributed to the inconsistent results across studies. In addition, this review did not apply prespecified minimum thresholds for sample size, intervention duration, or follow-up period; therefore, studies with relatively small samples or shorter intervention periods were pooled with larger or longer-duration trials, which may have contributed to clinical and methodological heterogeneity. The pooled findings should therefore be interpreted with caution. Second, the relatively small number of studies available for subgroup analyses, particularly when comparing the MCI and dementia populations, reduced the reliability of these findings. Publication bias was not assessed in some subgroup analyses because fewer than 10 studies were available. Although different dementia subtypes may present distinct cognitive characteristics, subgroup analysis was not feasible because of insufficient subtype-specific data. In addition, most studies have primarily focused on short-term outcomes, limiting their ability to evaluate the long-term sustainability of cognitive improvements. Many studies also relied on outcome measures that may lack the sensitivity needed to detect subtle yet clinically meaningful changes in cognitive domains, such as executive function.

Future studies should prioritize long-term studies to evaluate the sustainability of cognitive improvements and refine intervention designs to target executive functions more effectively. Furthermore, incorporating more sensitive and comprehensive outcome measures, such as the Alzheimer’s Disease Assessment Scale–Cognitive Subscale, will be essential for accurately assessing the impact of interventions. By addressing these gaps and optimizing intervention designs, clinicians and researchers can better harness the potential of digital technologies to mitigate cognitive decline in individuals with MCI or dementia.

## 5. Conclusions

This systematic review and meta-analysis demonstrated that DCIs significantly enhanced global cognitive function compared to usual care. However, the active control comparison remains inconclusive because it was based on a limited number of studies and may have been underpowered; therefore, this finding should not be interpreted as evidence of equivalence. Significant improvements were observed in individuals with MCI, highlighting the potential utility of this approach in addressing early cognitive decline. Nevertheless, these findings should be interpreted with caution owing to the heterogeneity of the included studies, the limited availability of subgroup data, and the generally low quality of evidence for several outcomes.

## Figures and Tables

**Table 1:** Clinical Questions (Population, Intervention, Control, and Outcomes).

Category	Description
Population	Participants diagnosed with mild cognitive impairment or dementia by specialized health professionals or based on standardized diagnostic criteria
Intervention	Digital technology-based interventions specifically designed to improve cognitive function, either independently or in combination with other interventions (such as physical activities, including exergaming)
Comparison	Active controls (non-digital interventions designed to improve cognitive function) Usual care (no intervention or non-specific interventions not specifically aimed at improving cognitive function)
Outcomes	Measures of global cognitive function, executive function, and quality of life

**Table 2:** Characteristics of studies.

Author(s) Year Country	Research Design	Participant Characteristics			Duration, Time, Frequency	Intervention Group	Control Group	Results
		Sample Size N (I/C)	Age (M ± SD)	Women N (%)				
Mild Cognitive Impairment								
Baldimtsi (2023) Greece [4]	RCT	122 (28/11/24 /31/28)	I: 66.1 (10.0) C_1_: 73.0 (8.5) C_2_: 71.0 (8.0) C_3_: 70.4 (7.4) C_4_: 74.4 (7.0)	I: 21 (75.0) C_1_: 5 (45.5) C_2_: 20 (83.3) C_3_: 29 (93.5) C_4_: 24 (85.7)	3 m (32 sessions) 2–3/wk 20–30 min	VR Exergaming (VRADA) VR Training System, which combines physical exercise (indoor cycling) with cognitive tasks (e.g., arithmetic, memory games) Provides an immersive virtual environment using a VR headset Includes motivational features such as real-time feedback, goal-setting, and progress tracking	4 Control groups Bike - Cycling only PE - Simple and complex physical exercises without cognitive components Mixed - Combines physical and cognitive exercises but not in a VR environment Non-contact control - No intervention	Global cognitive function (MMSE) Executive functions (TMT-B) Memory (RAVLT, WAIS-digit span forward) Depression and Anxiety (GDS) Functional abilities
Bevilacqua (2023) Italy [1]	RCT	62 (35/27)	I: 75.8 (8.1) C: 74.2 (8.1)	I: 17 (48.6) C: 15 (55.6)	3 m everyday	Computer-based cognitive intervention (RESILIEN-T) Tablet-based interface and wearable activity tracker (iHealth Wave) Provides content related to cognitive training, nutrition, physical activity, and social engagement	No intervention	Global cognitive function (MoCA) Memory (MAC-Q) QoL (EQ-5D-5L, QoL-AD) Psychological wellbeing (WEMWBS) Usability (SUS) Adherence to the Intervention
Chuang (2023) Taiwan [30]	RCT	80 (20/20 /20/20)	I_1_: 76.4 (9.8) I_2_: 76.2 (8.6) I_3_: 81.5 (7.0) C: 77.1 (9.3)	I_1_: 15 (75.0) I_2_: 16 (80.0) I_3_: 16 (80.0) C: 17 (85.0)	3–4 m (36 sessions) 2–3/wk 90 min	Computer-based cognitive intervention (BrainHQ) Computer-based cognitive training program targeting attention, memory, processing speed, and visual recognition Provides adaptive training with real-time feedback 3 Intervention groups COG - BrainHQ only SEQ - 45 min of physical training followed by 45 min of BrainHQ. SIMUL - dual-task training integrating cognitive tasks (BrainHQ) and physical exercises simultaneously	Physical training - Aerobic, resistance, and balance training.	Global cognitive function (MoCA) Memory (FR, WL, SS) IADL (Lawton IADL)
Manser (2023) Switzerland [19]	RCT	16 (10/6)	I: 79.9 (7.6) C: 73.7 (12.9)	I: 3 (30.0) C: 4 (66.7)	3 m (19–24 sessions) 5 times more/wk 21 min more	Computer-based Exergaming (Brain-IT training) Multi-domain exergaming combining physical exercise and cognitive training with heart rate variability Implemented using the Senso Flex exergaming platform, targeting key neurocognitive functions: learning and memory, executive function, attention, and visuospatial ability	Usual care - Medication - Physical and occupational therapy - Lifestyle modifications	Global cognitive function (Qmci) Executive function (PEBL trail-making test part B, TAP, HOTAP-A, PEBL digit span backward) Memory (WMS-IV-LM, PEBL digit span forward) Attention (PEBL trail-making test part A, TAP) QoL (QoL-AD) Depression and Anxiety (DASS-21) Feasibility Usability (SUS) Acceptance (EEQ, BREQ-e)
Wong (2023) Hongkong [31]	RCT	143	I_1_: N/A I_2_: N/A I_3_: N/A C: N/A	I_1_: N/A I_2_: N/A I_3_: N/A C: N/A	Center FL & Long-term FL - 6 m - 5/wk - 60 min Short-term FL - 3 m - 5/wk - 120 min Control - 3 m - 5/wk - 120 min	Computer-based cognitive intervention (Rosetta Stone Language Learning Software) Foreign language training to enhance cognitive functions 3 Intervention groups Center FL - in-person training at community centers using Rosetta Stone Short-term FL - mobile application-based training over 3 months Long-term FL - mobile application-based training over 6 months	Music listening using a mobile phone	Global cognitive function (ADAS-Cog) Memory (ARS) Naming ability (BNT) Attention (WDST forward and backward, ANT) Verbal fluency (CVFT) QoL (SF-12)
Park (2022a) Korea [32]	RCT	56 (28/28)	I: 71.9 (3.1) C: 72.0 (2.4)	I: 16 (57.1) C: 17 (60.7)	2 m (24 sessions) 3/wk 45 min	VR Cognitive Intervention Conducted in a VR environment simulating real-world navigation tasks Developed using the Unity game engine, operated on a desktop with joystick controls Provides real-time feedback on task performance	No intervention	Spatial cognition (WAIS-BDT) Memory (SVLT) Hippocampal Function Navigation Performance
Park (2022b) Korea [33]	RCT	32 (16/16)	I: 72.3 (5.1) C: 70.9 (4.5)	I: 7 (43.8) C: 10 (62.5)	2 m (16 sessions) 2/wk	VR Cognitive Intervention Virtual shopping training, which is conducted in a virtual supermarket environment using a tablet-based VR application Tasks focus on multiple cognitive domains, such as planning, problem-solving, memory, attention, and inhibitory control Provides real-time performance feedback	No intervention	Executive function (EFPT) IADL (IADL) Performance in Virtual Shopping Tasks
Kang (2021) Korea [6]	RCT	41 (23/18)	I: 75.5 (4.7) C: 73.3 (7.0)	I: 17 (73.9) C: 12 (66.7)	1 m (8 sessions) 2/wk 20–30 min	VR Cognitive Intervention Multidomain cognitive training in a fully immersive 3D VR environment Interactive tasks targeting attention, executive function, memory, and visuospatial skills Utilizes Oculus Rift CV1 headset and controllers Provides real-time feedback and interactive elements	Usual therapy - Pharmacotherapy	Global cognitive function (MMSE) Visuospatial function (RCFT) Executive function (COWAT, TMT-B, SCWT) Attention (TMT-A, digit span forward and backward test) Memory (SVLT) Language abilities (BNT) Depression and Anxiety (GDS, AES. PANAS) QoL (QoL-AD) SSQ
Han (2020) Korea [7]	RCT	24 (12/12)	I: 72.8 (7.9) C: 73.4 (6.0)	I: 7 (58.3) C: 4 (33.3)	10 wk (20 sessions) 2/wk 60 min	Computer-based Exergaming Song-based cognitive stimulation therapy Conducted with therapeutic musical devices, including percussion and keyboard-shaped instruments Tasks involve synchronized tapping with visual and auditory cues Offers cognitive stimulation on a desktop screen with moving lyrics and color-matching elements Provides real-time auditory and tactile feedback	Usual care	Global cognitive function (MMSE, MoCA) Disease severity (CDR-SOB) Depression (BDI) QoL (QoL-AD)
Manenti (2020) Italy [16]	RCT	49 (18/14/17)	I_1_: 75.3 (3.3) I_2_: 76.3 (4.9) C: 78.1 (4.1)	I_1_: 5 (27.8) I_2_: 10 (71.4) C: 10 (58.8)	Clinic-VRRS - 1 m (12 sessions) - 60 min Tele@H-VRRS - 3 m (36 sessions) - 3/wk - 60 min Tele@H-UCS - 3 m - (36 sessions) - 3/wk - 60 min Clinic-TAU - 1 m - (12 sessions) - 60 min	VR Cognitive Intervention (VRRS) Conducted on a computer-based platform with interactive cognitive tasks * Tasks target memory, visuospatial abilities, attention, and executive functions Use adaptive difficulty progression and real-time performance monitoring Three Intervention groups Clinic-VRRS + Tele@H-VRRS - face-to-face VRRS training followed by home-based VRRS Clinic-VRRS + Tele@H-UCS - face-to-face VRRS training followed by home-based, non-digital, unstructured cognitive activities	Clinic-TAU - Traditional face-to-face cognitive rehabilitation - Reality-oriented therapy, reminiscence therapy, cognitive strategy learning	Global cognitive function (MMSE) Executive function (TMT-B) Memory (RAVLT, FCSRT, EMQ, ROCFT-R) Attention (TMT-A) Visuoconstructional Skill (CDT) Language abilities (VFT, FPL, FPC, BADA) Depression (GDS_ Behavioral symptoms (NPI) QoL (QoL-AD) Usability (SUS)
Robert (2020) France [17]	RCT	46 (25/21)	I: 79.8 (7.0) C: 78.8 (6.6)	I: 10 (40.0) C: 14 (66.0)	3 m 1/wk 30 min	Computer-based cognitive intervention (MeMo) A web-based cognitive training application for personalized cognitive function enhancement Includes two training categories: memory (visual memory, working memory, and associative memory) and mental flexibility & attention (processing speed, inhibitory control, and reaction anticipation) Provides real-time performance tracking and adaptive difficulty levels	No intervention	Global cognitive function (MMSE) Executive functions (TMT-A, Stroop test, DSST, FAB) Memory (FCSRT) Behavioral symptoms (NPI, AI) Caregiver perception (IQCODE)
Thapa (2020) Korea [35]	RCT	68 (34/34)	I: 72.6 (5.4) C: 72.7 (5.6)	I: 28 (82.4) C: 24 (70.6)	2 m (24 sessions) 3/wk 100 min	VR Exergaming Immersive VR-based training conducted using Oculus Quest headsets and wireless hand controllers Tasks simulate real-world scenarios through interactive VR environments Educational program on general health care	Educational program on general health care	Global cognitive function (MMSE) Executive function (TMT-A, TMT-B, SDST) Brain function (EEG) Physical function (gait speed, 8-foot up-and-go test, hand grip strength) Adherence
Liao (2020) Taiwan [34]	RCT	34 (18/16)	I: 75.5 (5.2) C: 73.1 (6.8)	I: 11 (61.1) C: 12 (75)	3 m (36 sessions) 3/wk 60 min	VR Exergaming Conducted using the HTC Vive system, which includes VR glasses and wireless hand controllers Tasks simulate real-world scenarios, including navigating a metro station, grocery shopping, cooking Combines physical exercises (such as Tai Chi, resistance, aerobic activities) with cognitive tasks Provides real-time feedback and adaptive difficulty levels	Physical training - Stretching, aerobic exercise, and balance training Cognitive training - Word recall, simple arithmetic problems, and visual-spatial tasks	Global cognitive function (MoCA) Executive function (EXIT25) Memory (CVVLT) IADL (Lawton IADL) Brain activation (NIRS)
Li (2019) China [36]	RCT	141 (78/63)	I: 69.5 (7.3) C: 71.5 (6.8)	I: 33/84 C: 42/63	24 wk 3–4/wk 120–160 min	Computer-based cognitive intervention Training consists of eight tasks: visual working memory, 30-s memory, episodic memory, speed of calculation, visual search, alertness, mental rotation, and image re-arrangement tasks	No intervention	Global cognitive function (MMSE, ACE-revised) Memory (ROCF, AVLT-Huashan) Executive function (Sharp trail test-A and B, DSST, Stroop color–word test)
Liao (2019) Taiwan [37]	RCT	34 (18/16)	I: 75.5 (5.2) C: 73.1 (6.8)	I: 11 (61.1) C: 12 (75)	3 m (36 sessions) 3/wk 60 min	VR Exergaming Conducted using the Kinect system with VR glasses and hand controllers Tasks emphasize IADL-focused real-world scenarios, including navigating a metro station, grocery shopping, cooking Combines physical exercises (such as Tai Chi, resistance, and aerobic activities) with cognitive tasks Provided visual and auditory feedback	Physical training - Aerobic and resistance exercise and balance training Cognitive tasks integrated into functional exercises - Naming objects, solving arithmetic problems during walking, and reciting memory tasks	Executive function (TMT-A, TMT-B, SCWT) Gait performance Dual-Task Interference
Nousia (2019) Greece [3]	RCT	46 (25/21)	I: 71.2 (5.1) C: 71.9 (6.2)	I: 19 (76) C: 16 (76.2)	15 wk 2/wk 30–60 min	Computer-based cognitive intervention Conducted using RehaCom Cognitive Therapy software The training targets cognitive domains such as episodic and delayed memory, verbal memory, attention, processing speed, and executive function	Standard clinical care	Executive function (TMT-A, TMT-B, CDT) Memory (immediate word recall, word recognition, delayed word memory test) Verbal fluency (Semantic fluency measure) Attention (digit span forward and backward) Language abilities (BNT)
Yang (2019) Taiwan [38]	RCT	66 (33/33)	I: 75.4 (6.6) C: 81.7 (7.2)	I: 25 (75.8) C: 27 (81.8)	3 m (36 sessions) 3/wk 45 min	Computer-based cognitive intervention Virtual interactive working memory training conducted using CogniPlus software, an intelligent interaction system Intervention includes four working memory training modules: updating (visual memory), spatial encoding, rehearsal, and updating (spatial memory) Provides real-time performance feedback and adjusts task difficulty to participant’s ability	Training program using a tablet computer - Reading online e-books - Playing online games	Global cognitive function (MMSE, MoCA) Memory (Digit span backward, WMS-III, MMQ)
Damirchi (2018) Iran [45]	RCT	44 (11/13/11/9)	I_1_: 67.9 (3.8) I_2_: 67.8 (4.7) C_1_: 68.8 (3.7) C_2_: 69.7 (4.9)	I_1_: 26 (57.0) I_2_: 19 (43.0) C_1_: NA C_2_: NA	6 wk 3/wk 2–30 min	Computer-based cognitive intervention (Modified My Better Mind) Four different cognitive games include tasks designed to enhance visual attention, working memory, processing speed, verbal memory, reasoning, strategic planning, and spatial skills 2 Intervention groups Mental training (ME) - Computer-based cognitive training only ME + PT - Both physical and mental training, completing cognitive exercises before engaging in physical activities	2 Control groups Physical training (PT) - Aerobic exercise - Muscular strength - Range of movement exercises No intervention	Global cognitive function (WAIS-III) Executive function (Stroop test, digital span forward test, digit symbol coding test) Biomarker (BDNF, Irisin)
Delbroek (2017) Belgium [18]	RCT	20 (10/10)	I: 86.9 (5.6) C: 87.5 (6.6)	I: 8 (80) C: 5 (50)	6 wk 2/wk 18–30 min	Computer-based Exergaming (BioRescue) Combines balance exercises (including weight-shifting and obstacle navigation) with cognitive tasks (such as memory recall and decision-making) Tasks adapt to participants’ abilities and progressively increase in difficulty	Usual care - Continued usual daily activities - Standard physical therapy	Global cognitive function (MoCA) Physical function (Tinetti-POMA, iTUG, iTUG dual-task) Motivation (IMI) Emotional Response (OERS)
Han (2017) Korea [8]	RCT	42 (20/22)	I: 73.7 (4.8) C: 74.5 (6.4)	I: 10 (43.5) C: 10 (50)	1 m 2/wk 30 min	Computer-based Exergaming (USMART) A tablet-based, self-administered cognitive training program based on spaced retrieval training Tasks include spaced retrieval exercises, where participants recall words at increasing intervals and expand word sets upon consecutive correct recall Provides automated verbal instructions to guide participants	Usual care	Global cognitive function (MMSE) Memory (WLMT, WLRT, WLRcT, SMCQ) Depression (GDS)
Hagovská (2017) Slovakia [20]	RCT	60 (30/30)	I: 67.8 (6.5) C: 68.2 (4.2)	I: 18 (54) C: 13 (49)	10 wk (20 sessions) 30 min	Computer-based cognitive intervention (CogniPlus) The programs target attention, working memory, long-term memory, planning of everyday activities, and visual-motor abilities Involves five sub-programs: Alert, Nback, Names, Pland, and Vismo Combined with physical exercise in the sub-programs	Classical group cognitive training - Training targets verbal fluency, memory, psychomotor learning, communication, and reasoning	Global cognitive function (ACE) ADL (FAQ) Attention (Stroop test) QOL (Spitzer QoL)
Savulich (2017) England [21]	RCT	42 (21/21)	I: 75.2 (7.4) C: 76.9 (8.3)	I: 10 (47.6) C: 7 (33.3)	1 m (8 sessions) 60 min	Computer-based cognitive intervention A tablet-based intervention involves associating geometric patterns with spatial locations to improve episodic memory and visuospatial abilities Includes gamified elements such as stimulating music and progression rewards Provides Adaptive difficulty progression	Bingo Group - Played Bingo games, which do not target specific cognitive abilities on a tablet	Global cognitive function (MMSE) Memory (BVMT-R) Depression and Anxiety (GDS, AES) Reaction Time Motivational and Emotional Responses
Dimitriadis (2016) UK [22]	RCT	158 (53/50/55)	I: 69.7 (5.3) C_1_: 71.2 (3.9) C_2_: 66.4 (6.1)	I: 40 (75.5) C_1_: 40 (76) C_2_: 42 (76)	10 wk 4/wk 90 min	Computer-based cognitive intervention (Novel Serious game) A tablet-based application for mnemonic strategy training program (mini iPad tablet) Search for the hidden object in real space with real-time screenshot Provides a dual-task activity using hands and arms during the hide-and-seek task	2 Control groups Physical training (PT) - Watching YouTube documentaries No intervention	Global cognitive function (MMSE, CDR-sum of boxes) Memory (CVLT) Executive function (TMT-A, TMT-B) Attention (Digit span forward and backward) ADL (IADL) Depression (GDS) Brain function (EEG)
Lin (2016) USA [43]	RCT	21 (10/11)	I: 72.9 (8.2) C: 73.1 (9.6)	I: 5 (50) C: 5 (45.5)	6 wk 4/wk 60 min	Computer-based cognitive intervention (INSIGHT) Training consists of five tasks: eye for detail, peripheral challenge, visual sweeps, double decision, and target tracker	Mental leisure activities Online crossword, Sudoku, and solitaire games	Visual processing speed and attention (Useful Field of view) Executive function (EXAMINER) ADL (Timed IADL) Brain function (central executive network, default mode network)
Tarnanas (2014) Greece [23]	RCT	66 (32/34)	I: 70.5 (4.3) C: 70.9 (4.4)	I: 20 (62.5) C: 21 (61.8)	5 m 2/wk 90 min	VR Cognitive Intervention (Museum cognitive training) Conducted in an immersive VR environment, allowing to navigate and interact with museum exhibits Tasks include following instructions to locate items, listening to artifact stories and answering related questions, and performing actions depicted in historical exhibits	No intervention	Global cognitive function (MMSE) Memory (RAVLT, ROCFT) Executive function (TMT-B, SCWT, letter fluency) Visuoconstructional skills (ROCFC) Language abilities (BNT, CFT) Depression (GDS)
Zhu (2023) Taiwan [39]	Quasi- Experimental Study	69 (35/34)	I: 72.7 (6.5) C: 72.9 (5.8)	I: 30 (85.7) C: 22 (64.7)	2 m 2/wk 40 min	Computer-based Exergaming (HappyGoGo) Exergaming system with infrared motion sensing and a laptop computer Engaged in interactive movement-based games, requiring them to interact with projected images and complete exergaming tasks Exercise focused on upper and lower limb movements and Simulated ADL incorporating cognitive function-oriented tasks	Usual care	Global cognitive function (MoCA) Loneliness scale
Dementia								
Kim (2023) USA [44]	RCT	16 (8/8)	T: 82.2 (8.4)	T: 13 (81.3)	5 wk (10 sessions) 2/wk 30–40 min	VR Cognitive Intervention (Nature-Based VR) Conducted using Oculus Quest 2 VR headsets and the Nature Treks VR program Explore 11 themed virtual nature environments with interactive elements Provides varied locomotion options, including teleportation, free movement, snap rotation, and arm-swing navigation	Usual care - Facility-provided arts and crafts activities	QoL (QoL-AD) Depression (CSDD) Emotional health (OERS)
Rai (2021) England [24]	RCT	61 (31/30)	I: 74.0 (6.8) C: 71.8 (8.5)	I: 9 (29) C: 10 (33)	11 wk 2–3/wk 30 min	Computer-based Cognitive Intervention (iCST) Touchscreen tablet-based cognitive stimulation therapy application Includes 21 structured activities adapted from paper-based CST, with a mix of game-like features, audio-visual stimuli, and discussion prompts Encourages social interaction through joint activities	Usual care - Usual dementia treatments such as medication	Global cognitive function (ADAS-cog) QoL (QoL-AD, EQ-5D) Depression and Anxiety (CSDD, HADS) Behavioral symptoms (NPI) Functional abilities (BADLS) Dyadic Relationship Quality (QCPR) Usability (CUA) Computer User Self-Efficacy
Van Santen (2020) Netherland [25]	RCT	112 (73/39)	I: 79.0 (6.0) C: 79.0 (7.0)	I: 36 (49.0) C: 16 (41.0)	6 m 5/wk	Computer-based Exergaming Using interactive cycling systems (including DiFiets, Fietslabyrint, PraxFit, and SilverFit Mile) Participants cycled on a stationary bike while viewing simulated outdoor routes on a screen Implemented in psychogeriatric day care centers	Usual care - Regular day care center activity program	Global cognitive function (MMSE) Executive function (TMT-B, TMT-A) Physical function (SPPB, PASE, number of falls) Motivation (IMI) Psychological wellbeing Social functioning (GIP) Caregiver outcomes (EQ-5D-5L, NPI-Q, SSCQ)
Çinar (2020) Turkey [26]	RCT	60 (30/30)	I: 74.3 (7.6) C: 70.9 (7.8)	I: 16 (53.3) C: 14 (46.7)	3 m 7/wk 15–20 min	Computer-based Cognitive Intervention (BEYNEX) Includes three 5-min computer-based cognitive games daily targeting multiple cognitive domains Included a 3-min physical exercise video Training progress was tracked remotely by clinicians	No intervention	Global cognitive function (MoCA, CANTAB tests) ADL (Bayer ADL) Depression (GDS)
Karssemeijer (2019) Netherlands [2]	RCT	115 (38/38/39)	I: 79.0 (6.9) C_1_: 80.9 (6.1) C_2_: 79.8 (6.5)	I: 18 (47.3) C_1_: 17 (44.7) C_2_: 18 (46.2)	3 m 3/wk 30–40 min	Computer-based Exergaming Conducted using Bike Labyrinth, a stationary cycling system with an interactive video screen Participants cycled through digitally simulated environments while completing cognitive tasks Tasks included seven cognitive training levels, focusing on response inhibition, task switching, and processing speed, with progressively increasing difficulty	2 Control groups Aerobic Training - A stationary bike without cognitive engagement Relaxation and Flexibility Exercises - Non-intensive physical activity	Executive function (TMT-A, TMT-B, SCWT, Letter fluency) Memory (LLT, WAIS-III, Digit span, WMS-III)
Wiloth (2018) Germany [27]	RCT	99 (56/43)	I: 82.7 (6.2) C: 82.2 (5.3)	I: 39 (69.6) C: 32 (74.4)	10 wk 2/wk 90 min	Computer-based Exergaming (Physiomat®) Game training involves concurrent dual-task training of balance control with cognitive demands Tasks include follow-the-ball tasks and trail-making tasks	Motor Placebo Group - Physical engagement without targeting cognitive improvement	Global cognitive function (MMSE) Motor-cognitive task performance (Physiomat® performance) Physical function (TUG, POMA) Depression (GDS) Fall efficacy
De Luca (2016) Italy [28]	RCT	20 (10/10)	I: 78.0 (5.4) C: 77.8 (5.3)	I: 5 (50.0) C: 5 (50.0)	2 m (24 sessions) 3/wk 45 min	Computer-based Cognitive Intervention A computer-based platform using a web application Tasks include seven cognitive activities targeting praxis abilities, attention, visual-spatial memory, and verbal fluencies Activities involve moving objects, recalling and pairing images, copying figures, and word association	Conventional Cognitive Training - Traditional paper-and-pencil cognitive training	Global cognitive function (MMSE) Verbal fluency (CVF, LVF(a)) Attention (AM) Praxis abilities (CA) Dementia severity (BANSS) Depression (GDS) Behavioral symptoms (BPRS) ADL (IADL, basic ADL)
Yu (2015) China [40]	RCT	32 (16/16)	83 (70–99)	22 (69)	4–8 wk 1–2/wk (8 sessions) 30 min	Computer-based Exergaming Touchscreen video games requiring working memory and attention control Includes games such as Bingo, Connect the dot ultimate, Find difference, and Mosquito splash	Conventional cognitive training - Training elements were matched with the intervention training	Global cognitive function (MMSE) Neuropsychiatric symptom (NPI) Agitated behavior (Cohen-Mansfield Agitation Inventory) Depression (CSDD)
Lee (2013) Hong Kong [41]	RCT	19 (7/6/6)	T: 77.7 (6.1)	I: 6 (85.7) C_1_: 3 (50.0) C_2_: 4 (66.7)	6 wk 2/wk 30 min	Computer-based Cognitive Intervention (Computer-Assisted Errorless Learning Program) A touchscreen computer-based program with a touch-pen input device Errorless learning principles applied to memory training focus on sensory memory, working memory, and prospective memory Provides immediate positive feedback	2 Control groups Therapist-Led Errorless Learning Program - In-person using paper-based materials that include cognitive tasks Waiting-listing control - General cognitively challenging activities	Global cognitive function (MMSE, DRS) Memory (LLT, BAPM) ADL (MBI, Lawton IADL) Depression (GDS)
Mixed Population								
Diaz Baquero (2022) Spain [29]	RCT	75 (43/32)	I: 73.6 (6.0) C:N/A	I: 25 (59.3) C:N/A	4 m 2–3/wk 30–40 min	Computer-based Cognitive Intervention (GRADIOR) A touchscreen computer-based cognitive rehabilitation program including various exercises Tasks target orientation, attention, memory, executive function, perception, calculation, inference, and more Program content is customized to individual cognitive levels	Usual care	Global cognitive function (MMSE, ADAS-cog) Executive function (TMT-A, TMT-B, WAIS-III) Language (LVF(b), SVF) Visuospatial and Reasoning Abilities (CAMCOG, RBMT) Depression (GDS) QoL (EQ-5D-5L)
Yoo (2021) Korea [42]	RCT	24 (12/12)	I: N/A C: N/A	I: 8 (66.7) C: 8 (66.7)	5 wk 3/wk 30 min	Computer-based Cognitive Intervention (IPUZZLE) ICT-based visual perception training Arranging random puzzle pieces to match the target image	Traditional visual perception training	Global cognitive function (MMSE, MOCA) Visual Perception Function (MVPT-3, LOTCA-G) Feasibility

Note: * The study refers to this intervention as a “Virtual Reality Rehabilitation System,” but it is not a traditional VR-based training. Instead, it is a computer-based cognitive training program conducted on a screen, without the use of immersive VR technology. Abbreviations: ABC, Activities-Specific Balance Confidence Scale; ACE-III, Addenbrooke’s Cognitive Examination-III; ADAS, Alzheimer’s Disease Assessment Scale; ADAS-Cog, Alzheimer’s Disease Assessment Scale–Cognitive Subscale; ADKS, Alzheimer’s Disease Knowledge Scale; AI, Apathy Inventory; AM, Attentive Matrices; ANT, Attention Network Test; ASCOT, Adult Social Care Outcomes Toolkit; AVLT, Auditory Verbal Learning Test; BADA, Battery for the Analysis of Aphasic Deficits; BADL, Basic Activities of Daily Living; BADLS, Bristol Activities of Daily Living Scale; BANSS, Bedford Alzheimer Nursing Severity Scale; BAPM, Brief Assessment of Prospective Memory; BDI, Beck Depression Inventory; BDNF, Brain-Derived Neurotrophic Factor; BPRS, Brief Psychiatric Rating Scale; BVMT-R, Brief Visuospatial Memory Test-Revised; CA, Constructional Apraxia Test; CAMCOG, Cambridge Cognition Examination; CANTAB, Cambridge Neuropsychological Test Automated Battery; CDR-SOB, Clinical Dementia Rating Scale—Sum of Boxes; CSDD, Cornell Scale for Depression in Dementia; CUA, Computer Usability Assessment; CVF, Category Verbal Fluency; CVLT, California Verbal Learning Task; CVVLT, Chinese Version of the Verbal Learning Test; D-KEFS-TM, Delis–Kaplan Executive Function System Trail-Making Component; DASS-21, Depression, Anxiety, and Stress Scale-21; DHEA-S, Dehydroepiandrosterone Sulfate; DRS, Dementia Rating Scale; DSST, Digit Symbol Substitution Test; DQoL, Dementia Quality of Life; EAL, Experienced Autonomy List; EEQ, Exergame Enjoyment Questionnaire; EFPT, Executive Function Performance Test; EMQ, Everyday Memory Questionnaire; EQ-5D, Euro Quality of Life-5 Dimension; EQ-5D-5L, EuroQol 5-Dimension 5-Level; EVQ, Ecological Validity Questionnaire; EXIT-25, Executive Interview 25; FAB, Frontal Assessment Battery; FCSRT, Free and Cued Selective Reminding Test; FPL, Phonemic Verbal Fluency; FPC, Fluency Phonemic and Categorical; FR, Facial Recognition; GAD-7, Generalized Anxiety Disorder Scale; GAS, Goal Attainment Scale; GDS, Geriatric Depression Scale; GIP, Behavior Observation Scale for Intramural Psychogeriatrics; GSE, General Self-Efficacy Scale; HADS, Hospital Anxiety and Depression Scale; HOTAP-A, Higher-Order Test of Attentional Performance Picture-Sorting Test Part A; IMI, Intrinsic Motivation Inventory; IQCODE, Informant Questionnaire on Cognitive Decline in the Elderly; IADL, Instrumental Activities of Daily Living; LLT, Location Learning Test; LOTCA-G, Lowenstein Occupational Therapy Cognitive Assessment-Geriatric; LVF(a), Letter Verbal Fluency; LVF(b), Lexical Verbal Fluency; MAC-Q, Memory Assessment Clinics-Questionnaire; MMQ, Multifactorial Memory Questionnaire; MNA, Mini Nutritional Assessment; MMSE, Mini-Mental State Examination; MoCA, Montreal Cognitive Assessment; MVPT-3, Motor-Free Visual Perception Test-3; NPI, Neuropsychiatric Inventory; NPS, Net Promoter Score; OERS, Observed Emotions Rating Scale; PAL, Pleasant Activities List; PANAS, Positive and Negative Affect Schedule; PASE, Physical Activity Scale for the Elderly; PHQ-8, Personal Health Questionnaire-8; POMA, Performance-Oriented Mobility Assessment; QCPR, Quality of Co-Parenting Relationship; Qmci, Quick Mild Cognitive Impairment Screen; QoL, Quality of Life; QoL-AD, Quality of Life in Alzheimer’s Disease; QLQ-8, Quality of Life Questionnaire; RBMT, Rivermead Behavioral Memory Test; RBANS, Repeatable Battery for the Assessment of Neuropsychological Status; RCFT, Rey Complex Figure Test; ROCFT, Rey–Osterrieth Complex Figure Test; RAVLT, Rey Auditory Verbal Learning Test; SCWT, Stroop Color and Word Test; SCQ, Sense of Competence Questionnaire; SGDS, Short Form of the Geriatric Depression Scale; SMAS-30, Self-Management Ability Scale; SPPB, Short Physical Performance Battery; STS, Sit-to-Stand Test; SVF, Semantic Verbal Fluency; SVLT, Seoul Verbal Learning Test; SUS, System Usability Scale; TAMQ, Technology Acceptance and Motivation Questionnaire; TAP, Test of Attentional Performance; TMT-A, Trail-Making Test Part A; TMT-B, Trail-Making Test Part B; TUG, Timed Up-and-Go Test; VAT, Visual Attention Test; VEGF, Vascular Endothelial Growth Factor; WAIS-III, Wechsler Adult Intelligence Scale, Third Edition; WDS, Wechsler Digit Span Task; WEMWBS, Warwick–Edinburgh Mental Wellbeing Scale; WLMT, Word List Memory Test; WLRT, Word List Recall Test; WLRcT, Word List Recognition Test; WMS-III, Wechsler Memory Scale-Third Edition; WMS-IV-LM, Logical Memory Subtest of Wechsler Memory Scale-Fourth Edition.

## Data Availability

All data analyzed in this study are included in the published article and its Supplementary Materials and Table S2. Additional extracted data are available from the corresponding author upon reasonable request.

## Supplementary Materials

The following supporting information can be downloaded at: https://www.mdpi.com/article/10.3390/medicina62061162/s1, Supplementary S1. Detailed search strategy; Supplementary S2. Grading of Recommendation, Assessment, Development and Evaluation (GRADE) quality of evidence; Supplementary S3. Funnel plot of publication bias; Supplementary S4: PRISMA 2020 Checklist [52].

## References

[1] Bevilacqua R., Felici E., Cucchieri G., Amabili G., Margaritini A., Franceschetti C., Barboni I., Paolini S., Civerchia P., Raccichini A. (2023). Results of the Italian RESILIEN-T pilot study: A mobile health tool to support older people with Mild Cognitive Impairment. J. Clin. Med..

[2] Karssemeijer E.G., Aaronson J.A., Bossers W.J., Donders R., Olde Rikkert M.G., Kessels R.P. (2019). The quest for synergy between physical exercise and cognitive stimulation via exergaming in people with dementia: A randomized controlled trial. Alzheimer’s Res. Ther..

[3] Nousia A., Martzoukou M., Siokas V., Aretouli E., Aloizou A.-M., Folia V., Peristeri E., Messinis L., Nasios G., Dardiotis E. (2021). Beneficial effect of computer-based multidomain cognitive training in patients with mild cognitive impairment. Appl. Neuropsychol. Adult.

[4] Baldimtsi E., Mouzakidis C., Karathanasi E.M., Verykouki E., Hassandra M., Galanis E., Hatzigeorgiadis A., Goudas M., Zikas P., Evangelou G. (2023). Effects of Virtual Reality Physical and Cognitive Training Intervention On Cognitive Abilities of Elders with Mild Cognitive Impairment. J. Alzheimer’s Dis. Rep..

[5] Park H., Ha J. (2024). Effect of digital technology interventions for cognitive function improvement in mild cognitive impairment and dementia: A systematic review and meta-analysis. Res. Nurs. Health.

[6] Kang J.M., Kim N., Lee S.Y., Woo S.K., Park G., Yeon B.K., Park J.W., Youn J.-H., Ryu S.-H., Lee J.-Y. (2021). Effect of cognitive training in fully immersive virtual reality on visuospatial function and frontal-occipital functional connectivity in predementia: Randomized controlled trial. J. Med. Internet Res..

[7] Han E., Park J., Kim H., Jo G., Do H.K., Lee B.I. (2020). Cognitive Intervention with Musical Stimuli Using Digital Devices on Mild Cognitive Impairment: A Pilot Study. Healthcare.

[8] Han J.W., Son K.L., Byun H.J., Ko J.W., Kim K., Hong J.W., Kim T.H., Kim K.W. (2017). Efficacy of the Ubiquitous Spaced Retrieval-based Memory Advancement and Rehabilitation Training (USMART) program among patients with mild cognitive impairment: A randomized controlled crossover trial. Alzheimer’s Res. Ther..

[9] Lee Y.G., Kim G.H., Cho K.J., Kim G.W. (2022). Development of cognitive interventional therapy program for mild cognitive impairment: Preliminary study. J. Korean Geriatr. Psychiatry.

[10] Han H.J., Ko M.J., Park A., Cheun J., Nam Y., Kim T.H. (2024). Development of a digital multidomain lifestyle intervention for mild cognitive impairment: A pilot study on the feasibility and efficacy of cognitive training. Digit. Health.

[11] Chandler J., Cumpston M., Li T., Page M.J., Welch V. (2019). Cochrane Handbook for Systematic Reviews of Interventions.

[12] Page M.J., McKenzie J.E., Bossuyt P.M., Boutron I., Hoffmann T.C., Mulrow C.D., Shamseer L., Tetzlaff J.M., Moher D. (2021). Updating guidance for reporting systematic reviews: Development of the PRISMA 2020 statement. J. Clin. Epidemiol..

[13] Nejadghaderi S.A., Balibegloo M., Rezaei N. (2024). The Cochrane risk of bias assessment tool 2 (RoB 2) versus the original RoB: A perspective on the pros and cons. Health Sci. Rep..

[14] Balshem H., Helfand M., Schünemann H.J., Oxman A.D., Kunz R., Brozek J., Vist G.E., Falck-Ytter Y., Meerpohl J., Norris S. (2011). GRADE guidelines: 3. Rating the quality of evidence. J. Clin. Epidemiol..

[15] Luo D., Wan X., Liu J., Tong T. (2018). Optimally estimating the sample mean from the sample size, median, mid-range, and/or mid-quartile range. Stat. Methods Med. Res..

[16] Manenti R., Gobbi E., Baglio F., Macis A., Ferrari C., Pagnoni I., Rossetto F., Di Tella S., Alemanno F., Cimino V. (2020). Effectiveness of an innovative cognitive treatment and telerehabilitation on subjects with mild cognitive impairment: A multicenter, randomized, active-controlled study. Front. Aging Neurosci..

[17] Robert P., Manera V., Derreumaux A., Ferrandez Y., Montesino M., Leone E., Fabre R., Bourgeois J. (2020). Efficacy of a web app for cognitive training (MeMo) regarding cognitive and behavioral performance in people with neurocognitive disorders: Randomized controlled trial. J. Med. Internet Res..

[18] Delbroek T., Vermeylen W., Spildooren J. (2017). The effect of cognitive-motor dual task training with the biorescue force platform on cognition, balance and dual task performance in institutionalized older adults: A randomized controlled trial. J. Phys. Ther. Sci..

[19] Manser P., Poikonen H., de Bruin E.D. (2023). Feasibility, usability, and acceptance of “Brain-IT”—A newly developed exergame-based training concept for the secondary prevention of mild neurocognitive disorder: A pilot randomized controlled trial. Front. Aging Neurosci..

[20] Hagovská M., Dzvoník O., Olekszyová Z. (2017). Comparison of two cognitive training programs with effects on functional activities and quality of life. Res. Gerontol. Nurs..

[21] Savulich G., Piercy T., Fox C., Suckling J., Rowe J.B., O’Brien J.T., Sahakian B.J. (2017). Cognitive training using a novel memory game on an iPad in patients with amnestic mild cognitive impairment (aMCI). Int. J. Neuropsychopharmacol..

[22] Dimitriadis S.I., Tarnanas I., Wiederhold M., Wiederhold B., Tsolaki M., Fleisch E. (2016). Mnemonic strategy training of the elderly at risk for dementia enhances integration of information processing via cross-frequency coupling. Alzheimer’s Dement. Transl. Res. Clin. Interv..

[23] Tarnanas I., Tsolakis A., Tsolaki M. (2014). Assessing virtual reality environments as cognitive stimulation method for patients with MCI. Technologies of Inclusive Well-Being: Serious Games, Alternative Realities, and Play Therapy.

[24] Rai H.K., Schneider J., Orrell M. (2021). An individual cognitive stimulation therapy app for people with dementia and carers: Results from a feasibility randomized controlled trial (RCT). Clin. Interv. Aging.

[25] van Santen J., Dröes R.-M., Twisk J.W., Henkemans O.A.B., van Straten A., Meiland F.J. (2020). Effects of exergaming on cognitive and social functioning of people with dementia: A randomized controlled trial. J. Am. Med. Dir. Assoc..

[26] Çinar N., Şahiner T.A.H. (2020). Effects of the online computerized cognitive training program BEYNEX on the cognitive tests of individuals with subjective cognitive impairment and Alzheimer’s disease on rivastigmine therapy. Turk. J. Med. Sci..

[27] Wiloth S., Werner C., Lemke N.C., Bauer J., Hauer K. (2018). Motor-cognitive effects of a computerized game-based training method in people with dementia: A randomized controlled trial. Aging Ment. Health.

[28] De Luca R., Bramanti A., De Cola M.C., Leonardi S., Torrisi M., Aragona B., Trifiletti A., Ferrara M.D., Amante P., Casella C. (2016). Cognitive training for patients with dementia living in a sicilian nursing home: A novel web-based approach. Neurol. Sci..

[29] Diaz Baquero A.A., Perea Bartolomé M.V., Toribio-Guzmán J.M., Martínez-Abad F., Parra Vidales E., Bueno Aguado Y., van der Roest H.G., Franco-Martín M.A. (2022). Determinants of adherence to a “GRADIOR” computer-based cognitive training program in people with mild cognitive impairment (MCI) and mild dementia. J. Clin. Med..

[30] Chuang I.-C., Chiau H.-Y., Liao W.-W., Wu Y.-R., Chang C.-H., Wu C.-Y. (2023). Effects of computer-based cognitive training combined with physical training for older adults with cognitive impairment: A four-arm randomized controlled trial. Digit. Health.

[31] Wong P., Tsang S., Deng Z., Antoniou M. (2023). Foreign language training via mobile application to improve cognitive functions in patients with mild cognitive impairment: Abridged secondary. Hong Kong Med. J..

[32] Park J.H. (2022). Effects of virtual reality-based spatial cognitive training on hippocampal function of older adults with mild cognitive impairment. Int. Psychogeriatr..

[33] Park J.H. (2022). Does the virtual shopping training improve executive function and instrumental activities of daily living of patients with mild cognitive impairment?. Asian J. Psychiatr..

[34] Liao Y.-Y., Tseng H.-Y., Lin Y.-J., Wang C.-J., Hsu W.-C. (2020). Using virtual reality-based training to improve cognitive function, instrumental activities of daily living and neural efficiency in older adults with mild cognitive impairment. Eur. J. Phys. Rehabil. Med..

[35] Thapa N., Park H.J., Yang J.G., Son H., Jang M., Lee J., Kang S.W., Park K.W., Park H. (2020). The Effect of a Virtual Reality-Based Intervention Program on Cognition in Older Adults with Mild Cognitive Impairment: A Randomized Control Trial. J. Clin. Med..

[36] Li B.-Y., He N.-Y., Qiao Y., Xu H.-M., Lu Y.-Z., Cui P.-J., Ling H.-W., Yan F.-H., Tang H.-D., Chen S.-D. (2019). Computerized cognitive training for Chinese mild cognitive impairment patients: A neuropsychological and fMRI study. NeuroImage Clin..

[37] Liao Y.-Y., Chen I.-H., Lin Y.-J., Chen Y., Hsu W.-C. (2019). Effects of virtual reality-based physical and cognitive training on executive function and dual-task gait performance in older adults with mild cognitive impairment: A randomized control trial. Front. Aging Neurosci..

[38] Yang H.-L., Chu H., Kao C.-C., Chiu H.-L., Tseng I.-J., Tseng P., Chou K.-R. (2019). Development and effectiveness of virtual interactive working memory training for older people with mild cognitive impairment: A single-blind randomised controlled trial. Age Ageing.

[39] Zhu Y.Z., Lin C.F., Yang H.L., Jin G., Chiu H.L. (2023). Effects of exergaming on cognitive functions and loneliness of older adults with cognitive frailty. Int. J. Geriatr. Psychiatry.

[40] Yu R., Poon D., Ng A., Sit K., Lee J., Ma B., Lum C., Yeung F., Wong M., Hui E. Computer-assisted intervention using touch-screen video game technology on cognitive function and behavioural symptoms for community-dwelling older Chinese adults with mild-to-moderate dementia-preliminary results of a randomized controlled trial. Proceedings of the Special Session on How Can Digital Games Help Older Adults?.

[41] Lee G.Y., Yip C.C., Yu E.C., Man D.W. (2013). Evaluation of a computer-assisted errorless learning-based memory training program for patients with early Alzheimer’s disease in Hong Kong: A pilot study. Clin. Interv. Aging.

[42] Yoo K.-J.K., Kim J. (2021). Effect of visual perception training using ICT technology on visual perception and cognitive function of the elderly with dementia and mild cognitive impairment. J. Korea Aging-Friendly Ind. Assoc..

[43] Lin F., Heffner K.L., Ren P., Tivarus M.E., Brasch J., Chen D.G., Mapstone M., Porsteinsson A.P., Tadin D. (2016). Cognitive and neural effects of vision-based speed-of-processing training in older adults with amnestic mild cognitive impairment: A pilot study. J. Am. Geriatr. Soc..

[44] Kim J., Kim Y., Lee J., Oh S., Ory M. (2023). Efficacy of An Immersive Nature-based Virtual Reality Program on Depression, Emotional Health, and Qualityof-Life among Care Facility Residents with Alzheimer’s Disease (AD) and its Related Dementias (ADRD). Am. J. Health Behav..

[45] Damirchi A., Hosseini F., Babaei P. (2018). Mental training enhances cognitive function and BDNF more than either physical or combined training in elderly women with MCI: A small-scale study. Am. J. Alzheimer’s Dis. Other Dement..

[46] Kumar J., Patel T., Sugandh F., Dev J., Kumar U., Adeeb M., Kachhadia M.P., Puri P., Prachi F., Zaman M.U. (2023). Innovative approaches and therapies to enhance neuroplasticity and promote recovery in patients with neurological disorders: A narrative review. Cureus.

[47] Zhu S., Sui Y., Shen Y., Zhu Y., Ali N., Guo C., Wang T. (2021). Effects of Virtual Reality Intervention on Cognition and Motor Function in Older Adults with Mild Cognitive Impairment or Dementia: A Systematic Review and Meta-Analysis. Front. Aging Neurosci..

[48] Bang M., Kim M.A., Kim S.S., Kim H.S. (2024). Cognitive Training Using Virtual Reality: An Assessment of Usability and Adverse Effects. Arch. Rehabil. Res. Clin. Transl..

[49] Zuschnegg J., Schoberer D., Häussl A., Herzog S.A., Russegger S., Ploder K., Fellner M., Hofmarcher-Holzhacker M.M., Roller-Wirnsberger R., Paletta L. (2023). Effectiveness of computer-based interventions for community-dwelling people with cognitive decline: A systematic review with meta-analyses. BMC Geriatr..

[50] Sohn M., Yang J., Sohn J., Lee J.-H. (2023). Digital healthcare for dementia and cognitive impairment: A scoping review. Int. J. Nurs. Stud..

[51] Lee H., Lee S.H. (2023). Effectiveness of multicomponent home-based rehabilitation in older patients after hip fracture surgery: A systematic review and meta-analysis. J. Clin. Nurs..

[52] Page M.J., McKenzie J.E., Bossuyt P.M., Boutron I., Hoffmann T.C., Mulrow C.D., Shamseer L., Tetzlaff J.M., Akl E.A., Brennan S.E. (2021). The PRISMA 2020 statement: An updated guideline for reporting systematic reviews. BMJ.

