# Supplementary material for: Comparing Digital Cognitive Interventions to Active Controls and Usual Care for Mild Cognitive Impairment and Dementia: A Systematic Review and Meta-Analysis
Source: Medicina (Kaunas). 2026 Jun 15;62(6):1162. doi: 10.3390/medicina62061162 (PMC13304112; doi:10.3390/medicina62061162)
Supplement: Supplementary file 1 [file medicina-62-01162-s001.zip › medicina-4298014-Supplementary S1-S3.pdf]

Supplementary S1. Detailed search strategy

1) Ovid-Medline

1. exp Cognitive Dysfunction/
2. cognitive.mp.
3. (dysfunction* or impairment* or disorder* or deterioration*).mp.
4. 2 and 3
5. exp Dementia/
6. (dement* or Alzheimer*).mp.
7. mild cognitive impairment.mp.
8. 1 or 4 or 5 or 6 or 7
9. exp Telemedicine/
10. exp Telerehabilitaion/
11. exp Exergaming/
12. (digital health or digital therap* or digital intervention).mp.
13. (telemedicine or telerehab* or telehealth* or tele monitor or mobile intervention* or mobile health* or mobile therap* or mobile app* or mhealth* or ehealth* or virtual health* or online health* or computer-based health* or internet-based health* or web-based health* or exergram* or Xbox Kinect or Wii fit or ICT or (information and communications technology)).mp.
14. 9 or 10 or 11 or 12 or 13
15. 8 and 14

2) Embase

1. 'cognitive defect'/exp OR 'cognitive defect'
2. cognitive:ab,kw,ti
3. dysfunction*:ab,kw,ti OR impairment*:ab,kw,ti OR disorder*:ab,kw,ti OR deterioration*:ab,kw,ti
4. #2 AND #3
5. 'dementia'/exp
6. dement*:ab,kw,ti OR alzheimer*:ab,kw,ti
7. 'mild cognitive impairment':ab,kw,ti
8. #1 OR #4 OR #5 OR #6 OR #7
9. 'telemedicine'
10. 'telerehabilitation'
11. 'exergaming'
12. ((digital AND health OR digital) AND therap* OR digital) AND intervention*
13. telemedicine:ab,kw,ti OR telerehab*:ab,kw,ti OR telehealth*:ab,kw,ti OR 'tele monitor':ab,kw,ti OR (mobile:ab,kw,ti AND (intervention*:ab,kw,ti OR health*:ab,kw,ti OR therap*:ab,kw,ti OR app*:ab,kw,ti)) OR mhealth*:ab,kw,ti OR ehealth*:ab,kw,ti OR ((online:ab,kw,ti OR virtual:ab,kw,ti OR 'computer based':ab,kw,ti OR 'internet based':ab,kw,ti OR 'web based':ab,kw,ti) AND health*:ab,kw,ti) OR exergram*:ab,kw,ti OR 'xbox kinect':ab,kw,ti OR 'wii fit':ab,kw,ti OR ict:ab,kw,ti OR 'information and

communication technology':ab,kw,ti
14. #9 OR #10 OR #11 OR #12 OR #13
15. #8 AND #14

3) Cochrane library

1. (cognitive):ti,ab,kw
2. (dysfunction* or impairment* or disorder* or deterioration*):ti,ab,kw
3. #1 and #2
4. (dementia):ti,ab,kw
5. (dement* or Alzheimer*):ti,ab,kw
6. (cognitive dysfunction): ti,ab,kw
7. ("mild cognitive impairment"):ti,ab,kw
8. #3 or #4 or #5 or #6 or #7
9. (Telemedicine):ti,ab,kw
10. (Telerehabilitation):ti,ab,kw
11. (Exergaming):ti,ab,kw
12. (digital health OR digital therap* OR digital intervention*)
13. (telemedicine or telerehab* or telehealth* or (tele NEXT monitor) or (mobile NEXT intervention*) or (mobile NEXT health*) or (mobile NEXT therap*) or (mobile NEXT app*) or mhealth* or ehealth* or (virtual NEXT health*) or (online NEXT health*) or (Virtual NEXT health*) or (computer NEXT based NEXT health*) or (internet NEXT based NEXT health*) or (web NEXT based NEXT health*) or exergram* or "Xbox Kinect" or "Wii fit" or ICT or "information and communication technology"):ti,ab,kw
14. #9 or #10 or #11 or #12 or #13
15. #8 and #14

4) CINAHL

1. cognitive dysfunction
2. cognitive
3. dysfunction* or impairment* or disorder* or deterioration*
4. S2 AND S3
5. Dementia
6. dement* or Alzheimer*
7. mild cognitive impairment or mci
8. S1 OR S4 OR S5 OR S6 OR S7
9. Telemedicine
10. Telerehabilitation
11. Exergaming
12. digital health OR digital therap* OR digital intervention*
13. telemedicine or telerehab* or telehealth* or tele monitor or mobile intervention* or mobile health* or mobile therap* or mobile app* or mhealth* or ehealth* or virtual health* or online health* or Virtual health* or computer-based health* or internet-based health* or web-based

health* or exergram* or Xbox Kinect or Wii fit or ICT

14. S9 OR S10 OR S11 OR S12 OR 13
15. S8 AND S14

5) Web of Science

1. ti=(cognitive dysfunction) OR ab=(cognitive dysfunction) OR ak=(cognitive dysfunction)
2. ti=(cognitive) OR ab=(cognitive) OR ak=(cognitive)
3. ti=(dysfunction* OR impairment* OR disorder* OR deterioration*) OR ab=(dysfunction* OR impairment* OR disorder* OR deterioration*) OR ak=(dysfunction* OR impairment* OR disorder* OR deterioration*)
4. #2 and #3
5. ti=(dementia) OR ab=(dementia) OR ak=(dementia)
6. ti=(dement* OR Alzheimer*) OR ab=(dement* OR Alzheimer*) OR ak=(dement* OR Alzheimer*)
7. ti=(mild cognitive impairment or mic) OR ab=(mild cognitive impairment or mic) OR ak=(mild cognitive impairment or mic)
8. #1 or #4 or #5 or #6 or #7
9. ti=(Telemedicine) OR ak=(Telemedicine) OR ab=(Telemedicine)
10. ti=(Telerehabilitation) OR ak=(Telerehabilitation) OR ab=(Telerehabilitation)
11. ti=(Exergaming) OR ak=(Exergaming) OR ab=(Exergaming)
12. ti=(digital health OR digital therap* OR digital intervention*) OR ab=(digital health OR digital therap* OR digital intervention*) OR ak=(digital health OR digital therap* OR digital intervention*)
13. (((((((((((((((((ti=(telehealth*)) OR ti=(tele monitor)) OR ti=(mobile intervention*)) OR ti=(mobile health*)) OR ti=(mobile therap*)) OR ti=(mobile app*)) OR ti=(mhealth*)) OR ti=(ehealth*)) OR ti=(virtual health*)) OR ti=(online health*)) OR ti=(computer-based health*)) OR ti=(internet-based health*)) OR ti=(web-based health*)) OR ti=(exergram*)) OR ti=(Xbox Kinect)) OR ti=(Wii fit)) OR ti=(information and communications technology)) OR ti=(ICT)
14. (((((((((((((((((ab=(telehealth*)) OR ab=(tele monitor)) OR ab=(mobile intervention*)) OR ab=(mobile health*)) OR ab=(mobile therap*)) OR ab=(mobile app*)) OR ab=(mhealth*)) OR ab=(ehealth*)) OR ab=(virtual health*)) OR ab=(online health*)) OR ab=(computer-based health*)) OR ab=(internet-based health*)) OR ab=(web-based health*)) OR ab=(exergram*)) OR ab=(Xbox Kinect)) OR ab=(Wii fit)) OR ab=(information and communications technology)) OR ab=(ICT)
15. (((((((((((((((((ak=(telehealth*)) OR ak=(tele monitor)) OR ak=(mobile intervention*)) OR ak=(mobile health*)) OR ak=(mobile therap*)) OR ak=(mobile app*)) OR ak=(mhealth*)) OR ak=(ehealth*)) OR ak=(virtual health*)) OR ak=(online health*)) OR ak=(computer-based health*)) OR ak=(internet-based health*)) OR ak=(web-based health*)) OR ak=(exergram*)) OR ak=(Xbox Kinect)) OR ak=(Wii fit)) OR ak=(information and communications technology)) OR ak=(ICT)
16. #12 or #13 or #14 or #15
17. #9 or #10 or #11 or #16

18. #8 and #17

6) Psyinfo

1. tiab(cognitive dysfunction)
2. tiab(cognitive)
3. tiab(dysfunction* or impairment* or disorder* or deterioration*)
4. [S2] AND [S3]
5. tiab(Dementia)
6. tiab(dement* or Alzheimer*)
7. tiab(mild cognitive impairment or mci)
8. [S1] OR [S4] OR [S5] OR [S6] OR [S7]
9. tiab(Telemedicine)
10. tiab(Telerehabilitation)
11. tiab(Exergaming)
12. tiab(digital health OR digital therap* OR digital intervention*)
13. tiab(telemedicine or telerehab* or telehealth* or tele monitor or mobile intervention* or mobile health* or mobile therap* or mobile app* or mhealth* or ehealth* or virtual health* or online health* or Virtual health* or computer-based health* or internet-based health* or web-based health* or exergram* or Xbox Kinect or Wii fit or ICT
14. [S9] OR [S10] OR [S11] OR [S12] OR [S13]
15. [S8] AND [S14]

Supplementary S2. Grading of Recommendation, Assessment, Development and Evaluation (GRADE) quality of evidence

Table S1. GRADE quality of evidence for digital cognitive interventions compared with usual cares

Outcome	Number of participants (Studies)	Study design	Risk of bias	Inconsistency	Indirectness	Imprecision	Publication Bias	Certainty
Cognitive function[1]	1,125 (13)	RCT	Serious [a]	Serious [b]	Not Serious	Not Serious	Not serious	⊕⊕○○ Low
Executive function[2]	568 (7)	RCT	Not serious	Very serious [c]	Not Serious	Not Serious	-	⊕⊕○○ Low
Quality of Life[3]	188 (4)	RCT	Serious [a]	Not serious	Not Serious	Serious [d]	-	⊕⊕○○ Low

GRADE: grading of recommendation, assessment, development, and evaluation; RCT: randomized controlled trial.

Note: [1]The assessments of cognitive function utilized the Mini-Mental State Examination and Montreal Cognitive Assessment, [2] The assessments of executive function utilized Trail Making Test B, Wechsler Memory Scale-Spatial Span subtest, Executive Interview 25, and Executive Function Performance Test, [3]The assessments of quality of life used the Quality of Life in Alzheimer's Disease.

[a] Uncertainty (High Risk and Unclear Risk) was observed in Blinding and Allocation Concealment.

[b] The statistical heterogeneity is higher than 50%.

[c] The statistical heterogeneity is higher than 75%.

[d] The total number of participants was below the typical threshold of 300, and the confidence interval is wide, indicating some uncertainty in the effect estimate.

Table S2. GRADE quality of evidence for digital cognitive interventions compared with active controls

Outcome	Number of participants (Studies)	Study design	Risk of bias	Inconsistency	Indirectness	Imprecision	Publication Bias	Certainty
Cognitive function[1]	166 (5)	RCT	Not serious	Serious [a]	Not Serious	Serious [b]	-	⊕⊕○○ Low
Executive	182	RCT	Not serious	Not serious	Not Serious	Serious [b]	-	⊕⊕⊕○

function[2]	(5)							Moderate
Quality of Life[3]	35 (1)	RCT	Not serious	-	Not Serious	Very Serious [c]	-	⊕⊕○○ Low

GRADE: grading of recommendation, assessment, development, and evaluation; RCT: randomized controlled trial.

Note: [1]The assessments of cognitive function utilized the Mini-Mental State Examination and Montreal Cognitive Assessment, [2] The assessments of executive function utilized Trail Making Test B, Wechsler Memory Scale-Spatial Span subtest, Executive Interview 25, and Executive Function Performance Test, [3]The assessments of quality of life used the Quality of Life in Alzheimer's Disease.

[a] The statistical heterogeneity is higher than 50%.

[b] The total number of participants was below the typical threshold of 300, and the confidence interval is wide, indicating some uncertainty in the effect estimate.

[c] The total number of participants was very small, and the wide confidence interval, including both no effect and clinically significant effects, indicates very serious uncertainty in the estimate.

Supplementary S3. Funnel plot of publication bias

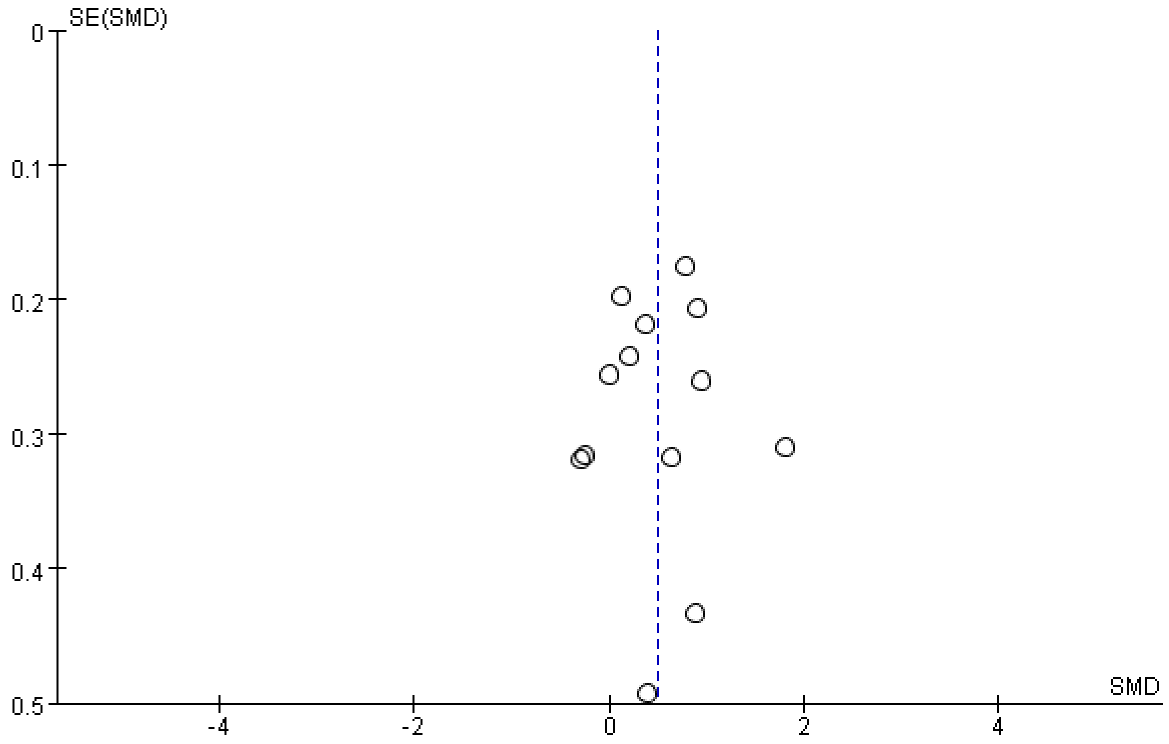

Figure S1. Funnel plot illustrating publication bias in the meta-analysis comparing digital cognitive intervention with usual care: global cognitive function
